# Radiomics for Diagnosis and Radiotherapy of Nasopharyngeal Carcinoma

**DOI:** 10.3389/fonc.2021.767134

**Published:** 2022-01-05

**Authors:** Yu-mei Zhang, Guan-zhong Gong, Qing-tao Qiu, Yun-wei Han, He-ming Lu, Yong Yin

**Affiliations:** ^1^Department of Oncology, The Affiliated Hospital of Southwest Medical University, Luzhou, China; ^2^Department of Radiotherapy, Shandong Cancer Hospital and Institute, Shandong First Medical University and Shandong Academy of Medical Sciences, Jinan, China; ^3^Department of Radiotherapy, People’s Hospital of Guangxi Zhuang Autonomous Region, Nanning, China

**Keywords:** nasopharyngeal carcinoma, computerized tomography, magnetic resonance imaging, positron emission computed tomography, radiomics

## Abstract

Nasopharyngeal carcinoma (NPC) is a malignant tumor of the head and neck. The primary clinical manifestations are nasal congestion, blood-stained nasal discharge, headache, and hearing loss. It occurs frequently in Southeast Asia, North Africa, and especially in southern China. Radiotherapy is the main treatment, and currently, imaging examinations used for the diagnosis, treatment, and prognosis of NPC include computed tomography (CT), magnetic resonance imaging (MRI), positron emission tomography (PET)-CT, and PET-MRI. These methods play an important role in target delineation, radiotherapy planning design, dose evaluation, and outcome prediction. However, the anatomical and metabolic information obtained at the macro level of images may not meet the increasing accuracy required for radiotherapy. As a technology used for mining deep image information, radiomics can provide further information for the diagnosis and treatment of NPC and promote individualized precision radiotherapy in the future. This paper reviews the application of radiomics in the diagnosis and treatment of nasopharyngeal carcinoma.

## 1 Introduction

Compared with other head and neck tumors, NPC has unique epidemiological, etiological, clinical, and genetic characteristics (1). According to the data of the International Agency for Research on Cancer, there are approximately 133,354 new cases of NPC, which accounts for only 0.7% of all cancers diagnosed in 2020. More than 70% of new cases occur in East and Southeast Asia, and South China is also an area with a high incidence. The age-standardized mortality rate in China is 1.6/100000, which is approximately twice that of NPC worldwide (2). Therefore, accurate treatment of NPC is imperative.

Because of the specific anatomical position and important adjacent structures of NPCs and the high sensitivity of NPC to radiotherapy, the main treatment for NPC is a comprehensive treatment based on radiotherapy. During radiotherapy, the medical images applied to NPC include magnetic resonance imaging (MRI), computed tomography (CT), positron emission tomography (PET)-CT, and PET-MRI (3). These various medical imaging methods have distinct characteristics. MRI has high contrast for different tissues, which provides high-resolution images of soft tissue; however, it requires a long acquisition time (4). CT has advantages in imaging bone and vascular invasion, and image acquisition is rapid; thus, it is well tolerated by patients (5, 6). In contrast to anatomical imaging, PET-CT combines biological metabolic information, and PET-MRI combines metabolic information with high-resolution soft-tissue images, and therefore, they are expected to become new methods for the diagnosis and treatment of NPC (5–7). These imaging methods have played a crucial role in target delineation, planning, quantitative evaluation, radiotherapy response tracking, and outcome and toxicity prediction of NPCs (8–11). However, the application of traditional images is aimed at diagnosing and treating diseases from a macro perspective. Patients with similar stages and grades of tumor experience different therapeutic effects with the same treatment due to the internal heterogeneity of the tumor (12). Only analyzing the disease from an anatomical level cannot meet the needs of treatment. With the increase in standards for radiotherapy, hidden information in images is valuable for improving NPC treatments. Radiomics is a technology that involves mining deep information in images, which has been used widely in the diagnosis, treatment, and prognosis of lung, esophageal, breast, rectal, and prostate cancers (13–16). There is an increasing number of studies investigating the diagnosis and treatment of NPC using radiomics. For example, one study applied metabolic information obtained from PET-CT to the treatment of head and neck squamous cell carcinoma with the aim of performing dose painting (17).

## 2 Radiomics

**Figure 1:**
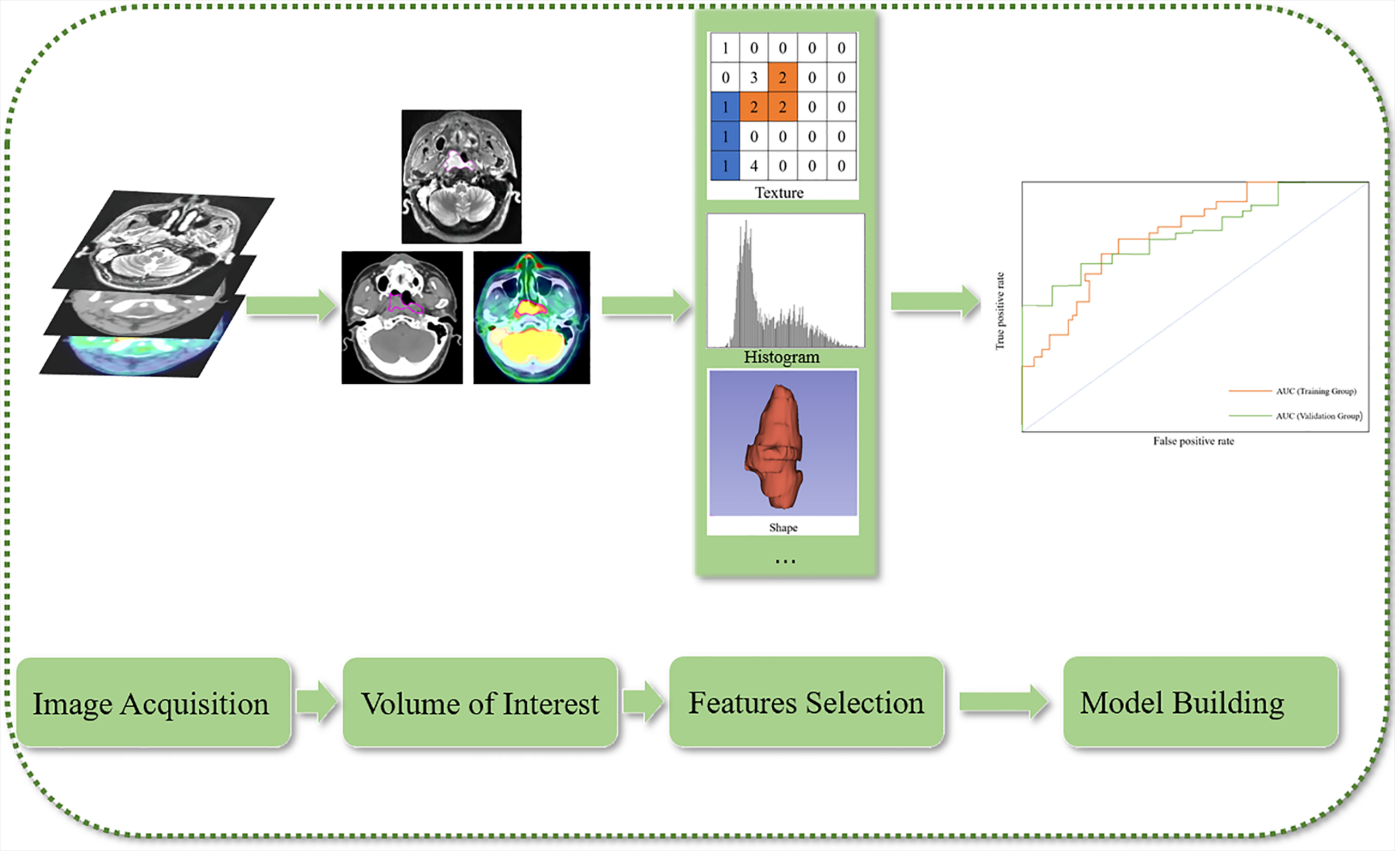
Flow chart of radiomics.

As an emergent field of transformational research, radiomics extracts quantitative features from medical images to decode the heterogeneity derived from tumor regions, metastatic lesions, and normal tissues, and explore microscopic changes in morphological and functional images (18). There are four steps in radiomics studies, which comprise image acquisition, tumor segmentation, feature extraction, and model development and validation (19) (**Figure 1**). Radiomics is distinct from traditional radiology, where images are not only interpreted visually; moreover, quantitative analyses are possible because the images are the data.

Radiomics statistical features can be divided into first-order, second-order, and high-order features. A first-order statistical feature describes the distribution of individual voxel values without considering the spatial relationship (20). Second-order features are usually described as ‘texture’ features; they describe the statistical correlation between voxels with similar (or dissimilar) contrast values and provide a measurement of intratumor heterogeneity. The high-order statistical method applies a filter grid to the image to extract repeated or non-repeated patterns (19). These data are combined with clinical data to develop models to improve the accuracy of diagnostic, treatment, and prognostic predictions. Mining image information and combining clinical medicine with engineering may become routine practice in the diagnosis and treatment of NPC in the future. Furthermore, radiomics will allow oncologists to establish relevant tumor databases and use this data to provide decision support for the diagnosis and treatment of tumors (21).

## 3 Radiomics Signature

When researchers make predictions about diagnosis or treatment based on radiomics, they need to first clarify the diseases and problems to be studied, and then collect relevant clinical data, such as hemoglobin, lymphocytes, etc. The features which are extracted from the volume of interest and related clinical parameters are filtered through various ways, such as Cox proportional hazards model and classifier, and then the final required model is established (22).

## 4 Application of Radiomics for the Diagnosis and Treatment of NPC

Most imaging studies on NPC have focused on MRI data, and few have reported on the use of CT, PET-CT, and PET-MRI images. Moreover, studies have focused predominantly on prognosis, and there is currently a lack of exploratory and prospective studies, even though retrospective studies can provide valuable clinical guidance for diagnosis, differential diagnosis, treatment, recurrence, and prognosis of the disease.

### 4.1 Diagnosis

#### 4.1.1 Diagnosis

The resolution of soft tissue on MRI is significantly superior to that of CT and PET-CT and can effectively show the range of parapharyngeal space, skull base, and intracranial tumors. It is the gold standard for the evaluation of NPC (5–7). NPC is prone to distant metastasis; therefore, PET-CT is now used to evaluate distant metastasis by providing systemic anatomical and metabolic information. The National Comprehensive Cancer Network guidelines (23) recommend MRI and PET-CT for the diagnosis of NPC. The TNM staging system (based on tumor size, regional lymph node involvement, and distant metastases) that is currently used to guide the diagnosis and treatment of NPC is regarded as the gold standard. However, during diagnosis and treatment, patients at similar stages have exhibited different treatment responses, which may be due to the internal heterogeneity of the tumor. Thus, Zhu et al. (24) developed a radiomics model that combined the features extracted from MRI data with clinical information to analyze the survival subgroups of early NPC (validation group C-index: 0.814), compared it with the T (C-index: 0.803) and TNM staging systems (C-index: 0.765), and concluded that performance of the radiomics model was superior to the TNM staging system. This may have a significant impact on individualized diagnoses, treatments, and prognoses of NPC in the future.

Compared with CT, MRI has a higher resolution of soft tissue, which is advantageous for imaging NPCs. Research on the application of CT combined with radiomics to the diagnosis and staging of disease remains limited. PET-MRI combines the metabolic characteristics of PET and the high-resolution characteristics of MRI (6). Compared with MRI, the increased [^18^F]-fluorodeoxyglucose (FDG) uptake of PET-MRI can better show the subtle changes in local lesions, and it can also provide a more suitable anatomical reference than can PET-CT (5). Chan et al. (25) found that the sensitivity, specificity, and accuracy for the diagnosis of primary tumors of head and neck MRI were 94.2%, 90.9%, and 99.5% (*p* = 0.75), respectively, 99.6%, 98.3%, and 99.2% (*p* = 0.92) for head and neck [^18^F]-FDG PET-CT, respectively, and 98.2%, 96.3%, and 99.3% (*p* = 0.87) for [^18^F]-FDG PET-MRI, respectively. The positive predictive value of PET-MRI in the diagnosis of distant metastasis (93.1%) is higher than that of MRI and PET-CT (78.8% and 83.3%, respectively). This was a prospective study that suggested that this imaging method has better diagnostic capabilities for nasopharyngeal cancer and may play an important role in the diagnosis and treatment of NPC in the future.

In a study of PET-MRI combined with radiomics, Feng et al. (26) developed a radiomics model of FDG PET-MRI and reported areas under the curve (AUC) of the training group based on T2-weighted imaging and PET models of 0.85 and 0.84, respectively, and those of the validation group of 0.83 and 0.82, respectively, which offers great promise for the clinical staging of NPCs. In terms of internal heterogeneity of tumors, Akram et al. (27) showed that the imaging features Neighboring Gray Tone Difference Matrix-busyness extracted from MRI data before and after treatment may reflect differences between recurrent and non-recurrent areas in tumors; moreover, they demonstrated the potential of radiomics in the identification of radiation resistance in tumors before treatment to select dose increments.

#### 4.1.2 Differential Diagnosis

Radiomics is advantageous not only for the diagnosis of diseases but also for differential diagnoses. The clinical manifestations and medical images of radiation-induced osteonecrosis and bone metastasis of the cervical spine are similar (28). Furthermore, the two conditions require different treatment methods and thus, require differentiation before treatment. The AUCs of MRI-based radiomics nomogram training and validation groups have been reported to be 0.725 and 0.720, respectively (28). Although CT and MRI are not applicable for differentiating between tumor recurrence and inflammation (29), PET-CT can distinguish between these two conditions; however, the high uptake of inflammation can affect the diagnosis of recurrence. The diagnostic performance of NPC images based on PET-CT imaging was evaluated using 42 cross combinations of six feature selection methods and seven classifiers. The optimal combination of feature selection and machine learning methods (the cross-combination fisher score [FSCR] + random forest [RF], FSCR + *k*-nearest neighborhood [KNN], FSCR + support vector machines [SVM] with radial basis function kernel [RBF-SVM], and minimum redundancy maximum relevance [MRMR] + RBF-SVM) to identify the two diseases were obtained (AUCs of 0.883, 0.867, 0.892, and 0.883, respectively; sensitivity: 0.833, 0.864, 0.831, and 0.750, respectively; specificity 1, 1, 0.873, and 1, respectively). Compared with the standard uptake value (SUV), total lesion glycolysis, and other indices, radiomics showed a higher AUCs (0.867–0.892 vs. 0.817), although the difference was not statistically significant (*p* = 0.462–0.560) (**Table 1**).

**Table 1:** Data of relevant models in references.

Purpose		Authors	Imaging	Application	Method/model	Results
Diagnosis	Diagnosis	Zhu et al. (24)	MRI	staging	support vector machine classifier	C-index : training&validation group : 0.827&0.814(based on model), 0.815&0.803(based on T), 0.842&0.756(based on TNM)
		Feng et al. (26)	PET-MRI	staging	logistic regression models	AUC: training group:0.84(PET),0.85(T2-weighted); validation group:0.82(PET),0.83(T2-weighted)
	Differential diagnosis	Zhong et al. (28)	MRI	cervical spine osteoradionecrosis and bone metastasis	nomogram model	AUC: training group : 0.725 ; validation group:0.720
		Du et al. (29)	PET-CT	recurrence and inflammation	7 types of machine learning classifiers	optimal combination of feature selection and machine learning methods
Treatment						
	Treatment response prediction	Yu et al. (30)	MRI	pretreatment prediction of adaptive radiation	logistic regression model	AUC: training group: 0.962(CET1-w), 0.895(T2-weighted),0.984(joint T1-T2); validation group:0.852(CET1-w), 0.750(T2-weighted),0.930(joint T1-T2)
		Zhao et al. (31)	MRI	predict the response to induction chemotherapy and survival	support vector machine, radiomics nomogram	C-index : training&validation group:0.952&0.863(radiomics signature with clinical data),0.708&0.549(clinical nomogram alone)
		Piao et al. (32)	MRI	early response of neoadjuvant chemotherapy	Cox regression model	AUC: 0.905(combined), 0.804(ClusterShade_angle135_offset 4)、0.762(Correlation_AllDirection_offshel_SD)
	Prognosis prediction					
		Ouyang et al. (33)	MRI	radiomics signature as a prognostic biomarker	multivariate Cox proportional hazards model	Hazard ratio (HR): 5.14(discovery set), 7.28(validation set)
		Shen et al. (34)	MRI	predicting progression-free survival (PFS)	Cox model	Model 5 incorporating radiomics, overall stage, and EBV DNA yielded the highest C-index for predicting PFS (training cohorts: 0.805, validation cohorts: 0.874)
		Ming et al. (35)	MRI	disease free-survival (DFS), overall survival (OS), distant metastasis-free survival (DMFS)	Cox regression model	C-index : validation group: 0.751(DFS)、0.845(OS)、0、643(DMFS)
		Yang et al. (36)	MRI	PFS	Nomogram	C-index: validation group: 0.811(including three factors),0.613(just TNM)
		Lv et al. (37)	PET-CT	PFS	Cox regression model	C-index: validation group: 0.67–0.73
		Xu et al. (38)	PET-CT	PFS	Cox’s proportional hazard model	C-index: 0.69(S3), 0.58(whole tumor)
		Zhang et al. (39)	MRI	distant metastasis	logistic regression model	AUC: training&validation groups: 0.827&0.792
		Zhang et al. (40)	MRI	local recurrence	Cox proportional hazard model, nomogram	C-index: validation groups: 0.74(radiomic features and multiple clinical variables)
		Raghavan et al. (41)	MRI	recurrence	multivariate logistic regression model, Cox proportional model	local recurrence model: 0.82(AUC), 0.73(sensitivity), and 0.74(specificity);distant metastasis model: 0.92(AUC), 0.79(sensitivity), and 0.84(specificity)
		Zhang et al. (42)	MRI	optimal machine-learning methods for the radiomics-based prediction of local failure and distant failure	machine-learning methods	optimal combination random forest + random forest AUC:0.8464 ± 0.0069
		Li et al. (43)	MRI	recurrence patterns	machine-learning methods,support vector machine (SVM) models	NPCs with in-field recurrences (NPC-IFR) and NPCs with non-progression disease (NPC-NPD) could be differentiated (AUCs: 0.727–0.835).
	Prediction of side effects	Liu et al. (44)	CT	prediction of Acute Xerostomia	support vector regression	accuracy: 0.9220, sensitivity: 100%
		Zhang et al. (45)	MRI	radiation-Induced Brain Injury	Random forest method	AUC: validation groups: 0.830 (model1), 0.773 (model2), and 0.716(model3)
	Stability characteristic study	Liang et al. (46)	MRI	Moddicom (v. 0.51),Pyradiomics (v. 2.1.2)	Spearman’s rank correlation	Selection of stable features of the disease is key.
		Lu et al. (47)	PET-CT	different contrast agents	ICC	features extracted from [11C] choline are more stable than those extracted from the [18F]-FDG contrast agent.
		Yang et al. (48)	PET-MRI	robust radiomic features	intraclass correlation coefficient (ICC) and spearman correlation coefficient	voxel size: 0.5 × 0.5 × 1.0 mm3; normalized grey level:64 and 128
		Lv et al. (49)	PET-CT	robustness	ICC	poor absolute-scale robustness still has good diagnostic performance.

### 4.2 Treatment

#### 4.2.1 Treatment Response Prediction

Radiotherapy is the main treatment for NPC during the early stage and radiotherapy and chemotherapy are the primary treatments during in late stage (6). For patients with intensity-modulated radiotherapy, weight loss, tumor regression, and other factors can result in large dose errors when applying the originally planned irradiation (30). In such cases, adaptive radiotherapy may be a better treatment option. Adaptive radiotherapy is usually administered to patients during radiation therapy, and the processes of imaging, sketching, and planning are repeated. The current radiotherapy system presents a significant economic burden for patients, and treatment is time-consuming and labor-intensive. Patients who need adaptive radiotherapy should be identified before treatment to improve treatment response. Yu et al. (30) used tumor marker features in MRI images acquired before treatment, and feature modeling (using enhanced T1 and T2 images provided AUCs of the enhanced T1, T2, and combined model verification groups of 0.852, 0.750, and 0.930, respectively), which may offer a basis for determining patient eligibility for adaptive radiotherapy and developing personalized treatments to reduce dose error. For the treatment response of NPC patients with advanced local progression to induction chemotherapy, Zhao et al. (31) developed a nomogram that combined multi-sequence MRI features before treatment with clinical parameters to predict the treatment effect in non-epidemic NPC areas. The model (training and validation group C-index: 0.952 vs. 0.863) had better predictive ability than the model developed using clinical parameters alone (training and validation group C-index: 0.708 vs. 0.549). In addition, in a study by Piao et al. (32), the AUC of the combined model was highest (AUC: 0.905) with the separate modeling of ClusterShade_angle135_offset 4 and Correlation_AllDirection_offshel_SD features based on enhanced magnetic resonance sequence imaging (AUC: 0.804 and 0.762, respectively). The combined model of the two features can help to determine the sensitivity and drug resistance in patients undergoing neoadjuvant chemotherapy, which is crucial for treatment scheme selection and treatment plan modification in patients with NPC.

#### 4.2.2 Prognosis Prediction

Most imaging studies have focused on the prognosis of NPC. These studies (33–36) demonstrate the effectiveness of conventional MRI in evaluating progression-free survival (PFS), disease-free survival, and overall survival in patients with NPC, in combination with clinical information, such as lymph nodes, Epstein-Barr virus, and tumor stage, which can guide personalized treatment selection and improve the quality of care. Ouyang et al. (33) calculated and analyzed the Radscore and found it can predict PFS as a biomarker. Shen et al. (34) developed five models based on different combinations of data: model 1: clinical data; model 2: overall staging; model 3: radiomics; model 4: radiomics + overall staging; and model 5: radiomics + overall staging + EB virus). Model 5 had a high C-index for predicting PFS (training group 0.805, validation group 0.874). Yang et al. (36) suggested a nomogram integrating lymph node, Dose Volume Histogram signature, reflecting planning score and TNM stage, that had a C-index of 0.811 for the prediction of PFS, which showed better performance than using TNM alone (C-index: 0.613). Furthermore, another study (37) used different combinations of PET, CT, and relevant clinical data to develop models and found that the model combining all three factors had the highest prediction performance (C-indices of the training and validation cohorts were 0.71–0.76 and 0.67–0.73, respectively). Another found that subregional radiomics analysis of NPC outperformed the whole tumor (C-index, 0.69 vs. 0.58) and the traditional AJCC (American Joint Committee on Cancer) staging system for PFS prediction (38).

Radiomics can predict not only treatment effects but also recurrence before treatment, which can improve treatment decision-making. For the prediction of recurrence, most studies use MRI images. Zhang et al. (39) developed models based on MRI radiomics to predict distant metastasis (AUCs of the training and validation groups: 0.827 and 0.792, respectively) and divided patients into low- and high-risk groups based on a risk cutoff score of 0.37 to indicate the risk of metastasis and determine the treatment strategy. A subsequent study (40) introduced a nomogram to radiomics to study local recurrence and found that the nomogram (C-index: 0.74) predicted recurrence more accurately than did radiomics and clinical variables (C-index: 0.59). The study by Raghavan et al. (41) preferred the prediction model, which not only predicted recurrence but also emphasized whether recurrence would occur in the form of local or distant metastasis. The AUC, sensitivity, and specificity of the local recurrence model were 0.82, 0.73, and 0.74, respectively, whereas those of the model for predicting distant metastasis were 0.92, 0.79, and 0.84, respectively. In addition, another study (42) combined machine learning with features extracted from MRI and applied different feature selection and classifier methods to determine the optimal combination (random forest + random forest), which laid a foundation for future studies of local recurrence and distant metastasis prediction combining MRI features with relevant clinical information. Li et al. (43) used radiomics with machine learning to analyze the radiation resistance of local recurrence (artificial neural network: 0.812; KNN:0.775; SVM: 0.732) using existing MRI data, which provided quantitative and objective evaluations of patients with NPC without requiring additional radiation exposure. Furthermore, NPCs with in-field recurrences could be differentiated from NPCs (AUCs: 0.727–0.835).

#### 4.2.3 Prediction of Side Effects

Radiomics can also be applied to the prediction of radiotherapy reactions the following radiotherapy for NPC. In a study of patients with acute xerostomia after radiotherapy (44), parotid CT images and saliva volume were acquired before, during, and after treatment to develop a model to predict changes in saliva volume after early radiotherapy (accuracy: 0.9220, sensitivity: 100%). The difference between the statistical and real values can then be used to predict the degree of dry mouth by predicting the amount of saliva. The diagnosis of radiation-induced brain injury in NPC mainly depends on MRI; however, MRI has limited use for early diagnoses and can only be used to evaluate morphological changes in late radiation-induced brain injury in the temporal lobe. Radiomics can examine microscopic characteristics, which can be used as markers as a basis for the treatment of early brain injury. Zhang et al. (45) developed three models combining machine learning and MRI radiomics; the AUCs of the validation group were 0.830 (95% confidence interval [CI]: 0.823–0.837), 0.773 (95% CI: 0.763–0.782), and 0.716 (95% CI: 0.699–0.733), respectively, which offers promise for applying radiomics to the study of related complications.

#### 4.2.4 Stability Characteristic Study

Because there are numerous methods to extract radiomics features, obtaining robust features is vital for the generalizability of radiomics models. Liang et al. (46) used two different feature extraction tools to extract features from different NPC MRI sequences. Different extraction methods had varying effects on the features, which may impact model development. Thus, the selection of stable features of the disease is key. In a study on PET-CT radiomics characteristics under different contrast agents, Lu et al. (47) selected [^18^F]-FDG and [^11^C] choline to examine segmentation and discretization and revealed that discretization has a greater impact on features than does segmentation, and features extracted from [^11^C] choline are more stable than those extracted from the [^18^F]-FDG contrast agent. Yang et al. (48) evaluated the reproducibility of features extracted from PET-MRI and found that a voxel size of 0.5 × 0.5 × 1.0 mm^3^ in PET, T2, and diffusion-weighted imaging data and a larger bin size allow the acquisition of stable characteristics. Although these studies focused on the definition and mode of feature generation, Lv et al. (49) analyzed the robustness of feature matrix parameters and found that poor absolute-scale robustness retained good diagnostic performance (**Table 1**).

## 5 Future

Artificial intelligence technologies and radiomics will be applied in the diagnosis and treatment of NPC in the field of target delineation, dose evaluation, plan design, outcome prediction, to realize the individualized clinical adaptive precision radiotherapy. However, there is still a significant gap between research and clinical application, which requires relevant modeling to not only meet or even exceed the industry gold standard but also solve some medical ethical problems (20). At present, many studies on radiomics are focused on NPC. However, radiomics may be extended to diseases other than tumors in the future and provide a reference for the majority of patients by establishing databases and other measures. In addition, radiomics can reduce medical costs and makes full use of medical image data to reduce injuries caused by invasive punctures; relevant models can solve problems of treatment and prognosis to save on medical costs and realize individualized treatment.

## 6 Conclusion

Multimodal imaging combined with radiomics offers new opportunities and methods for studying the diagnosis, treatment, and prognosis of NPC. The combination of radiomics and machine learning assists in the diagnosis and treatment of NPC. However, machine learning in radiomics is primarily applied to model selection. Although radiomics has numerous unique advantages, it also carries significant challenges, such as the need for big datasets for tumor model development, data sharing between different medical institutions, and various imaging protocols. Considerable progress is still needed to apply radiomics models to clinical practice. Future developments of radiomics require further forward-looking research and applications to promote individualized and intelligent treatment.

## Author Contributions

Y-MZ designed the study and wrote this paper. G-ZG, H-ML, Q-TQ, Y-WH, and YY conceived the study and revised the paper. All authors contributed to the article and approved the submitted version.

## Funding

This work was supported by the National Science Foundation of Shandong Province (ZR2020MH227) and the Guangxi Key Research and Development Plan (AB17195005).

## Conflict of Interest

The authors declare that the research was conducted in the absence of any commercial or financial relationships that could be construed as a potential conflict of interest.

## Publisher’s Note

All claims expressed in this article are solely those of the authors and do not necessarily represent those of their affiliated organizations, or those of the publisher, the editors and the reviewers. Any product that may be evaluated in this article, or claim that may be made by its manufacturer, is not guaranteed or endorsed by the publisher.
